# Assessment of cytotoxicity exerted by leaf extracts from plants of the genus *Rhododendron* towards epidermal keratinocytes and intestine epithelial cells

**DOI:** 10.1186/s12906-015-0860-8

**Published:** 2015-10-15

**Authors:** Ahmed Rezk, Alaa Al-Hashimi, Warren John, Hartwig Schepker, Matthias S. Ullrich, Klaudia Brix

**Affiliations:** Department of Life Sciences and Chemistry, Jacobs University Bremen, Campus Ring 1, D-28759 Bremen, Germany; Stiftung Bremer Rhododendronpark, Deliusweg 40, D-28359 Bremen, Germany

**Keywords:** *Rhododendron*, Bio-active compounds, Cytotoxicity, Mitochondrial activity, Programmed cell death

## Abstract

**Background:**

*Rhododendron* leaf extracts were previously found to exert antimicrobial activities against a range of Gram-positive bacteria. In this study, we investigated which of the extracts with these antimicrobial properties would be best suited for further exploitation. Specifically, the project aims to identify biologically active compounds that affect bacterial but not mammalian cells when applied in medical treatments such as lotions for ectopic application onto skin, or as orally administered drugs.

**Methods:**

Different concentrations of DMSO-dissolved remnants of crude methanol *Rhododendron* leaf extracts were incubated for 24 h with cultured epidermal keratinocytes (human HaCaT cell line) and epithelial cells of the intestinal mucosa (rat IEC6 cell line) and tested for their cytotoxic potential. In particular, the cytotoxic potencies of the compounds contained in antimicrobial *Rhododendron* leaf extracts were assessed by quantifying their effects on (i) plasma membrane integrity, (ii) cell viability and proliferation rates, (iii) cellular metabolism, (iv) cytoskeletal architecture, and (v) determining initiation of cell death pathways by morphological and biochemical means.

**Results:**

Extracts of almost all *Rhododendron* species, when applied at 500 μg/mL, were potent in negatively affecting both keratinocytes and intestine epithelial cells, except material from *R. hippophaeoides* var. *hippophaeoides*. Extracts of *R. minus* and *R. racemosum* were non-toxic towards both mammalian cell types when used at 50 μg/mL, which was equivalent to their minimal inhibitory concentration against bacteria. At this concentration, leaf extracts from three other highly potent antimicrobial *Rhododendron* species proved non-cytotoxic against one or the other mammalian cell type: Extracts of *R. ferrugineum were* non-toxic towards IEC6 cells, and extracts of *R. rubiginosum* as well as *R. concinnum* did not affect HaCaT cells. In general, keratinocytes proved more resistant than intestine epithelial cells against the treatment with compounds contained in *Rhododendron* leaf extracts.

**Conclusions:**

We conclude that leaf extracts from highly potent antimicrobial *R. minus* and *R. racemosum* are safe to use at 50 μg/mL in 24-h incubations with HaCaT keratinocytes and IEC6 intestine epithelial cells in monolayer cultures. Extracts from *R. rubiginosum* as well as *R. concinnum* or *R. ferrugineum* are applicable to either keratinocytes or intestinal epithelial cells, respectively. Beyond the scope of the current study, further experiments are required to identify the specific compounds contained in those Rhododendron leaf extracts that exert antimicrobial activity while being non-cytotoxic when applied onto human skin or gastrointestinal tract mucosa. Thus, this study supports the notion that detailed phytochemical profiling and compound identification is needed for characterization of the leaf extracts from specific *Rhododendron* species in order to exploit their components as supplementary agents in antimicrobial phyto-medical treatments.

**Electronic supplementary material:**

The online version of this article (doi:10.1186/s12906-015-0860-8) contains supplementary material, which is available to authorized users.

## Background

Plant extracts are commonly used in formulations of alternative and traditional medicine such as skin lotions, or when used as ingredients in dietary treatments and teas [1]. Plant-based medications are well-accepted by patients and are often preferred over chemically produced therapeutics because of their well-known health-benefitting bio-active ingredients [2–6]. Moreover, plant-extractable compounds have also gained a lot of attention in conventional medicine. For instance, plant-based drugs are now used for therapeutic treatment of diseases such as cancer and various inflammatory disorders [7, 8]. Therefore, knowing and assessing the potentials of plant-derived bio-active compounds is important for further drug development. This notion is deducible from the increasing interest of the pharmaceutical industry in gaining the rights to identify and exploit plant-borne compounds from species-rich rainforests in countries of tropical and subtropical regions [9–11]. While there is certainly a great potential in identifying plant-derived medication, the challenges associated with this venture must also be noted. Some of the current discussion revolving around this topic are: the protection of bio-diversity, acceptance of intellectual property rights, as well as biosafety of application [12, 13]. The aim of this study is to establish and provide an experimental, cell biological platform that allows for the identification of plant species that should be characterized and assessed in more detail.

So far, roughly 6 % of all higher plant species existing worldwide have been, or are currently being, assessed for their medicinal potential. In fact, only a minor proportion of these plant species have actually been subjected to detailed phytochemical profiling [14–16]. Bio-active compounds must first be purified before they can be assessed and eventually tested in clinical trials. Of course, the overall aim of the tests would be to ensure the efficacy of the biomolecules in particular therapeutic approaches. Simultaneously, drug safety and absence of undesirable side-effects are of the highest concern [17]. These considerations are important, regardless of whether pure compounds or crude extracts of an entire plant, or parts thereof, are used for the production of a pharmaceutically applicable plant ingredient [18].

The genus *Rhododendron*, comprising the species-richest group of wooden plants, belongs to the family *Ericaceae* and encompasses about one thousand species: the majority of which are indigenous to Asia [19]. In ethno-medicine, extracts of *Rhododendron* have been used traditionally in treating various disorders such as inflammatory conditions, common symptoms of cold, gastrointestinal disorders, skin diseases, or as pain killers [20]. Recent research highlighted that *Rhododendron* leaf extracts might be highly potent and beneficial to health due to properties they contain, such as anti-bacterial [21, 22], anti-allergic, and anti-inflammatory [23, 24] agents. The reported usefulness of crude extracts of *R. ferrugineum* and *R. anthopogon* [20, 25–27] is most likely due to the presence of terpenoids in high concentrations [25].

Previously, we investigated leaf extracts of 120 different *Rhododendron* species for their efficacy as antimicrobials in killing a variety of Gram-positive and Gram-negative bacteria [25]. In the current study, extracts of 12 of the *Rhododendron* species with highest anti-bacterial potencies were applied in different concentrations to monolayer cultures of human HaCaT epidermal keratinocytes and rat intestine epithelial cell line IEC6. Intestinal epithelial cells and keratinocytes are considered to be among the first points of contact when drugs are administered orally or applied ectopically, respectively. In general, bio-active compounds are considered cytotoxic when they alter cellular morphology or metabolism, interfere with the cytoskeleton or cell adhesion, affect cell proliferation rates or cell differentiation processes, or initiate programmed cell death [28]. Different cell types might exhibit differential responses towards a specific compound or plant extract. Consequently, it is neither sufficient to use only one cell line nor to apply just a single cytotoxicity assay in any safety assessment study.

The aim of this study was to assess possible cytotoxic effects of antimicrobial *Rhododendron* leaf extracts on mammalian cells in order to identify a potential candidate species for further analysis of safe use. Thus, the study contributes to on-going investigations on the bioactivity potential of plant species such as the *Rhododendron*. Hence, the effects of *Rhododendron* leaf extracts on cell survival, metabolism, and growth as well as on different cellular structures were monitored *in vitro* by an array of cell biological assays employing differentiated cell lines.

## Methods

### Collection of plant material and leaf extract preparation

Fresh leaf material of reliably identified *Rhododendron* species was used in this study (Table 1). The material was collected from January 2012 to December 2013 from plants grown in the Rhododendron-Park Bremen (www.rhododendronparkbremen.de). Each plant species was sampled once without considering seasonal variations. The identities of the plant species used in this study (Table 1) have been verified by reference to the German Gene Bank Rhododendron Database provided by the *Bundessortenamt* (www.bundessortenamt.de/rhodo) [25]. Material from all plant species used is publicly and freely available from the Rhododendron-Park Bremen upon request.

**Table 1:** List of *Rhododendron* species from which leaves were collected and used to prepare extracts that were screened for exhibiting cytotoxicity towards intestine epithelial cell cultures and monolayers of keratinocytes

Genebank-No.^a^	Species Name	Section	Sub-section
100.345	*R. ferrugineum* L.	Rhododendron	Rhododendron
100.007	*R. ambiguum* Hemsley	Rhododendron	Triflora
NA	*R. anthopogon* Don ssp. *anthopogon* Betty Graham	Pogonanthum	-
NA	*R. hirsutum* L.	Rhododendron	Rhododendron
100.326	*R. concinnum* Hemsley	Rhododendron	Triflora
100.322	*R. cinnabarinum* Hooker	Rhododendron	Cinnabarina
NA	*R. racemosum* Franchet	Rhododendron	Scabrifolia
100.404	*R. rubiginosum* Franchet	Rhododendron	Heliolepida
100.474	*R. xanthostephanum* Merrill	Rhododendron	Tephropepla
100.370	*R. minus* Michaux	Rhododendron	Caroliniana
100.392	*R. polycladum* Franchet	Rhododendron	Lapponica
100.353	*R. hippophaeoides* var. *hippophaeoides* Hutchinson	Rhododendron	Lapponica

^a^Gene bank numbers used in the collection of the Rhododendron-Park Bremen

*NA* Not a plant of the German Gene Bank Rhododendron but a verified plant of the Rhododendron-Park Bremen

Leaf material was frozen in liquid nitrogen and powdered using a KSW 3307 mill (Clatronic, Kempen, Germany). Crude extracts were prepared by soaking two grams of *Rhododendron* leaf powder in 10 mL of 80 % methanol for 24 h at 4 °C with constant shaking. Insoluble material was removed by centrifugation at 3,220 *g* for 30 min at 4 °C, and supernatants were stored at -20 °C for further use. Methanol was evaporated from the extracts using a Micro Modulyo lyophilizer (Edwards, Crawley, UK). Stock solutions were prepared by dissolving the residues in 100 % dimethyl sulfoxide (DMSO) (Carl Roth, Karlsruhe, Germany). Prior to the *in vitro* assays, the samples were mixed with the respective cell culture medium such that the final concentration of DMSO did not exceed 0.5 % (v/v), and 5, 50, or 500 μg of lyophilized powder per mL culture medium were applied to confluent IEC6 and HaCaT cell monolayers.

### Cell culture

The normal rat small intestine epithelial cell line IEC6 [29, 30] and the human keratinocyte cell line HaCaT [31, 32], purchased from the European Collection of Cell Cultures (Salisbury, UK), were used throughout this study. IEC6 cells were grown in Dulbecco’s modified Eagle’s Medium (DMEM High Glucose) (Lonza Group, Basel, Switzerland) supplemented with 10 % fetal calf serum (FCS) (Perbio Science, Bonn, Germany) and 10 μg/mL insulin (Sigma-Aldrich, Steinheim, Germany). IEC6 cells were incubated at 37 °C in a 5 % CO_2_ atmosphere in an incubator (Heraeus, Osterode, Germany). HaCaT cells were cultured in DMEM containing 10 % FCS and incubated at 37 °C in an 8.4 % CO_2_ atmosphere. Cell cultures were passaged once per week. All experiments were performed with cultures at approx. 70 % and 95 % confluence for IEC6 and HaCaT cells, respectively.

### Determination of cell viability and proliferative activity by MTT assays

Effects of *Rhododendron* leaf extracts on the viability and proliferative activity of cultured IEC6 and HaCaT cells were quantitated using the 3-(4,5-dimethylthiazol-2-yl)-2,5-diphenyltetrazolium bromide (MTT) assay (Carl Roth). This test is indicative for mitochondrial NADH-dependent dehydrogenase activity, which is proportional to both cell viability and proliferation rates of treated cultures [33–35]. A total of 1 × 10^4^ cells/well were seeded in single wells of 96-well plates (Greiner, Essen, Germany) and upon reaching the desired confluence, the cells were incubated with three different concentrations of *Rhododendron* leaf extracts (5, 50, and 500 μg per mL culture medium, not exceeding 0.5 % DMSO in content) for 24 h in complete medium and at standard culture conditions. Incubation of cells with culture medium containing DMSO at a final concentration of 0.5 % (v/v) was used as a negative control. Culture supernatants containing free-floating dead cells were removed at the end of the incubation period, replaced with fresh culture medium containing MTT at a final concentration of 0.5 % (w/v). The cell layers were then further incubated for another four hours. Subsequently, culture supernatants were removed, the cells adherent to the plate surface were collected in 100 % DMSO and incubated for 15 min at 37 °C to terminate the reaction and to dissolve formazan crystals. The absorbance of formazan formed by *Rhododendron* leaf extract-treated and non-treated control cell cultures was quantified at 595 nm in a microplate reader against the solvent (Tecan Group, Männedorf, Switzerland). Percentages of cell viability were calculated from triplicates using Eq. (1):

1 $$ \%\kern0.5em  of\kern0.5em  cell\kern0.5em  viability=\left(\frac{absorbance\kern0.5em  of\kern0.5em  treated\; cell s}{absorbance\kern0.5em  of\kern0.5em  control\kern0.5em  cell s}\right)\times 100 $$

### Propidium iodide staining of nuclei in cells with ruptured plasma membranes

The two cell lines were grown on cover glasses in 24-well Bio-One Cellstar plates (Greiner) to reach the desired degree of confluence. Next, cells were incubated with three different concentrations of *Rhododendron* leaf extracts (i.e. 5, 50, or 500 μg/mL) for 24 h as described above. Subsequently, cells were washed three times with phosphate-buffered saline (PBS) before being incubated for 45 min in 2 mg/mL propidium iodide (PI) (Carl Roth) and 5 μM Draq5™ (Biostatus, Leicester, UK) in culture medium at 37 °C. After washing three times in PBS, cells were fixed in 4 % paraformaldehyde (PFA) (Carl Roth) in 200 mM HEPES (pH 7.4) at room temperature for 20 min. Cells on cover glasses were washed again in PBS and distilled water before mounting them in Mowiol for subsequent laser scanning microscopy as described previously [36]. PI is not capable of penetrating cells with intact plasma membranes, however, if plasma membrane integrity is lost, PI gains access to the nucleus and forms complexes with the DNA. In contrast, Draq5™ serves as a nuclear counter-stain that transverses the intact plasma membrane and can therefore be used to determine the total cell number. Special care had to be taken when analyzing total cell numbers, because some plant leaf extracts could have exhibited anti-adhesive effects such that total cell numbers were significantly diminished after washing steps. Therefore, total cell numbers were determined and reported herein as a measure for anti-adhesive properties of *Rhododendron*-derived compounds.

### Phalloidin staining of the filamentous actin cytoskeleton

IEC6 and HaCaT cells were grown on cover glasses in 24-well plates to reach 70 % and 95 % confluence, respectively, and exposed to *Rhododendron* leaf extracts for 24 h as described above, while 0.5 % DMSO was used as a negative control. Cells were washed three times with PBS before fixation in 4 % PFA in 200 mM HEPES (pH 7.4) at room temperature for 20 min. After fixation, cells were washed with PBS before applying 0.2 % Triton X-100 in PBS for 5 min at room temperature, followed by several washing steps in PBS. Finally, cells were stained for 30 min at room temperature with a mixture of 3 μM FITC-labeled phalloidin (Sigma Aldrich) and 5 μM Draq5™ in PBS, the latter used as a counter-stain of nuclear DNA. Cover glasses were mounted in Mowiol for subsequent inspection by laser scanning microscopy (see below).

### MitoTracker® Red CMXRos staining of the mitochondrial matrix

Cells were incubated and treated as described above, before washing twice in phenol red-free HEPES-buffered culture medium for 5 min. Subsequently, the cells were incubated with phenol red-free culture medium containing 20 mM HEPES and 500 nM MitoTracker® Red CMXRos (Molecular Probes, Oregon, USA) for 45 min at 37 °C followed by several washes. The fluorescent dye accumulates in the mitochondrial matrix only when an intact membrane potential, due to active cellular metabolism, is present across the inner mitochondrial membrane. Cells were fixed with 4 % PFA in 200 mM HEPES (pH 7.4) for 20 min at room temperature, rinsed, and mounted on microscope slides as described above for subsequent microscopic inspection.

### Microscopy techniques

Stained cells were visualized with an LSM 510 confocal laser scanning microscope (Carl Zeiss, Jena, Germany) at excitation wavelengths of 488 nm, 543 nm and 633 nm for fluorophore excitation to visualize FITC-phalloidin, PI or MitoTracker® Red CMXRos, and Draq5™, respectively. Scans at a resolution of 1024 x1024 pixels were taken in the line averaging mode and at a pinhole setting of one airy unit. Color coding and image analysis was performed by using the LSM 510 software, release 3.2 (Carl Zeiss).

### Caspase-3 activity assay

For IEC6 cells, induction of apoptosis upon incubation with *R. ferrugineum* and *R. cinnabarinum* leaf extracts at the highest concentration, i.e. 500 μg/mL, was evaluated at different time intervals ranging from 1 to 24 h. The apoptosis assay was performed using the EnzChek Caspase-3 assay kit (Invitrogen, Karlsruhe, Germany) detecting activation of procaspase-3 and other Asp-Glu-Val-Asp (DEVD)-specific proteases. Lysates of treated IEC6 cells and non-treated controls were prepared according to the manufacturer’s protocol. Following clearance by centrifugation, the samples were incubated with 5 mM Z-DEVD-R110 substrate for 30 min at 4 °C. Lysates of IEC6 cells treated for 4 h at 37 °C with apoptosis-inducing staurosporin (10 mM) (Sigma-Aldrich) were used as positive controls, whereas no treatment or incubation with the solvent served as negative controls. Additionally, staurosporin-treated cells incubated with 1 mM of Ac-DEVD-CHO for 10 min served as a negative control since caspase-3 activity is blocked under these conditions. The extent of procaspase-3 activation was determined by fluorescence of liberated rhodamine upon excitation at 496 nm and reading the emission at 520 nm, using a microplate reader (Tecan Group, Männedorf, Switzerland). The values were normalized to equal amounts of DNA in the pellets after lysis, as determined by the Burton assay [37].

### Determination of minimum inhibitory concentrations

The minimum inhibitory concentration (MIC) was defined as the lowest concentration of *Rhododendron* leaf extract that inhibits visible growth of microorganisms after overnight incubation. The MIC was determined by a two-fold dilution assay in Mueller-Hinton broth (MHB) (Becton Dickinson, Heidelberg, Germany). The *Bacillus subtilis* strain *S168* was tested against 12 *Rhododendron* crude extracts (Table 1) [25]. All tests were performed in triplicates following the National Center for Clinical Laboratory Standards recommendations [38].

### Statistical evaluation

All assays were performed in triplicates and repeated at least three times in independent experiments unless stated otherwise. All data were expressed as means ± standard deviation (SD), as determined by using Origin software (MicroCal Software, Northampton, USA). The profile map shown in Fig. 9 was created using R (RStudio, Boston, USA). Levels of significance were calculated by One-Way ANOVA, and *p* < 0.05 was considered statistically significant. CellProfiler software [39] was used to determine total cell numbers (Draq5™-positive cells) *versus* numbers of dead cells (PI-positive cells). This software was also employed to quantify the MitoTracker® Red CMXRos and FITC-phalloidin fluorescence signal intensities as previously described by us [32].

## Results

### Classification of Rhododendron species based on antibacterial activities

In order to group the 12 selected *Rhododendron* species [25] according to their antibacterial activities, minimum inhibitory concentration (MIC) tests were conducted against *B. subtilis* . Accordingly, the plant species were classified into four major groups: six *Rhododendron* species formed the group with the highest antibacterial activity with an MIC of 50 μg/mL: *R. minus*, *R. racemosum*, *R. ferrugineum*, *R. rubiginosum*, *R. anthopogon* ssp. *anthopogon*, and *R. concinnum*. Another three species formed the group with moderately active extracts, with an MIC of 100 μg/mL: *R. cinnabarinum*, *R. hirsutum*, and *R. ambiguum*. The remaining *Rhododendron* species exhibited lower antibacterial activities with *R. xanthostephanum* and *R. polycladum* having an MIC of 150 μg/mL and *R. hippophaeoides* var. *hippophaeoides* requiring 300 μg/mL to efficiently produce an inhibition zone for *B. subtilis*.

### Cell viability and proliferation rates as quantified by the MTT assay

**Fig. 1:**
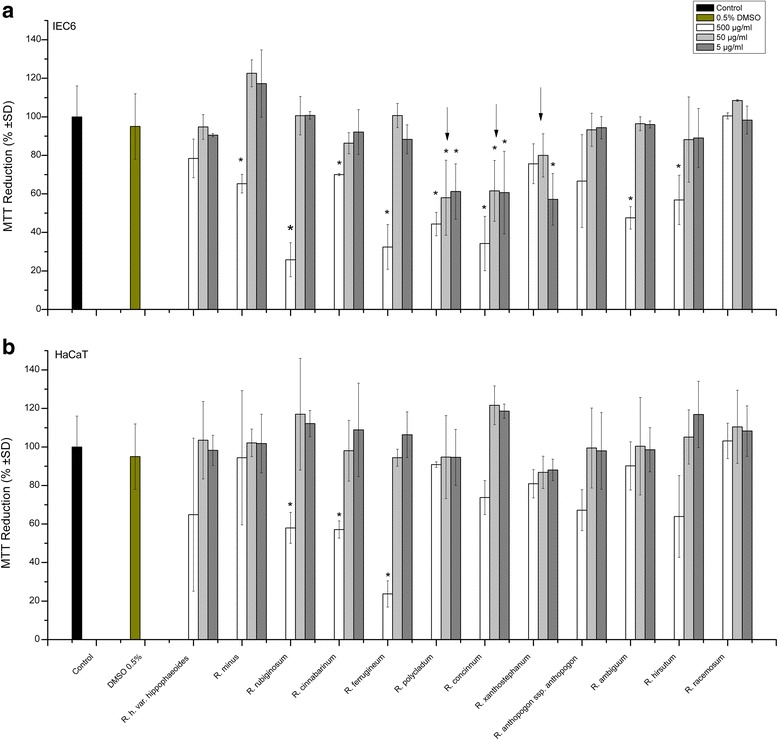
Effects of *Rhododendron* leaf extracts on IEC6 (**a**) and HaCaT (**b**) cells incubated with three different concentrations (5, 50, and 500 μg/mL) of leaf extracts as indicated. Cell viability and proliferation was analyzed by the MTT assay upon incubation at 37 °C for 24 h. The percentage of MTT reduction for each extract concentration was normalized to that of the 0.5 % DMSO solvent. Values are given as mean ± standard deviations from three independent experiments, each performed in triplicates. Statistical evaluation was performed by one way ANOVA-analysis; levels of significance are indicated as *for *p < 0.05*

The effects of leaf extracts prepared from 12 different *Rhododendron* species on cell viability and proliferation rates were initially estimated with the help of the MTT assay, as this test allows for a rapid screening of many samples. To this end, three different concentrations of *Rhododendron* leaf extract (5, 50, and 500 μg/mL) were applied to IEC6 and HaCaT cells for 24 h. As demonstrated in Fig. 1, incubation with extracts applied at lower concentrations (5 and 50 μg/mL) revealed no detectable change in MTT conversion rates of HaCaT cells, while the leaf extracts from *R. polycladum*, *R. concinnum*, and *R. xanthostephanum* affected IEC6 cellular metabolism negatively (arrows) in comparison to cells that were not treated at all, or treated with 0.5 % DMSO, suggesting that even 5 μg/mL of these extracts caused cytotoxic effects on intestine epithelial cells. *Rhododendron* leaf extracts used at the higher concentration of 500 μg/mL were more effective in reducing the MTT conversion ability of the treated cells (Fig. 1). Extracts of *R. rubiginosum*, *R. cinnabarinum*, and *R. ferrugineum* exerted cytotoxic effects as deduced from the significant decrease in the ability of both IEC6 and HaCaT cells to reduce MTT. In addition, samples from *R. minus*, *R. polycladum*, *R. concinnum*, *R. ambiguum*, and *R. hirsutum* induced statistically significant reductions in the MTT conversion ability of IEC6 cells, but not HaCaT cells. Interestingly, treatments with leaf extracts of *R. hippophaeoides* var. *hippophaeoides*, *R. anthopogon* ssp. *anthopogon*, and *R. racemosum* did not exhibit any significant alterations in metabolic activities or cell viability highlighting their potential non-cytotoxicity (Fig. 1).

### Analysis of plasma membrane integrity

**Fig. 2:**
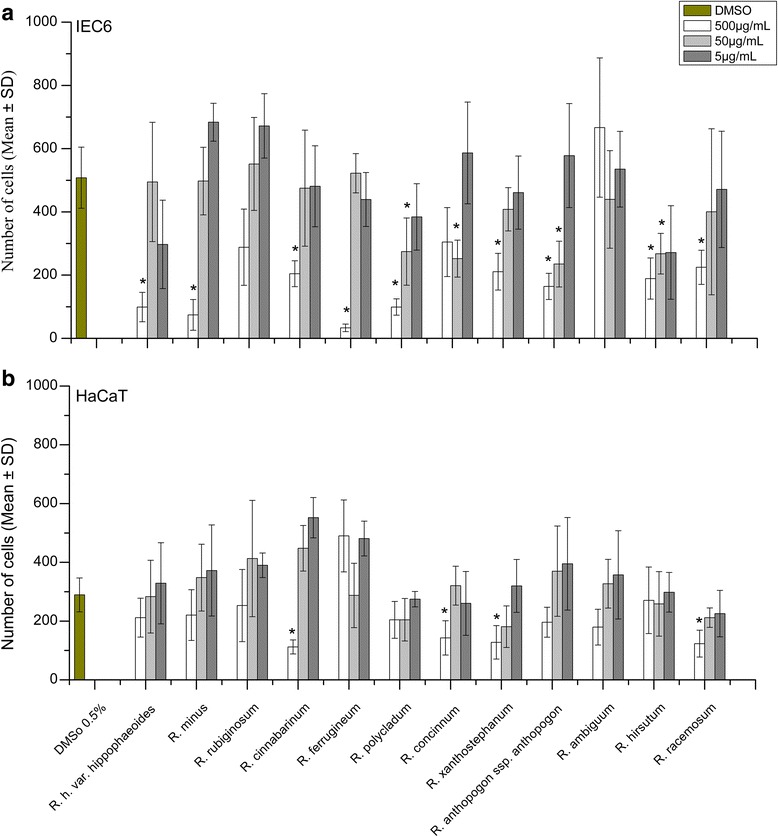
Effects of *Rhododendron* leaf extracts on the cell numbers of IEC6 (**a**) and HaCaT (**b**) cells after 24 h incubation at 37 °C with three different concentrations (5, 50, and 500 μg/mL) of leaf extracts as indicated. The total number of cells as determined by Draq5™ staining reflects the effects of leaf extracts on cell viability and adhesion since only monolayer-associated cells were stained and counted in this assay. Values are given as mean ± standard deviations from three independent experiments, each performed in triplicates. Statistical evaluation was performed by one way ANOVA-analysis; levels of significance are indicated as *for *p < 0.05*

In order to verify the initially observed cytotoxicity of the *Rhododendron* leaf extracts on IEC6 and HaCaT cell lines, changes in the integrity of the plasma membrane of the cells upon incubation with leaf extracts were tested. For this, PI acquisition was assayed, which occurs only in those cells that feature ruptured plasma membranes. Cell staining with Draq5™ allowed an estimation of the total cell number. The results summarized in Table 2, Fig. 2, and Additional file 1: Figure S1 demonstrated that incubation of IEC6 cells with 5 and 50 μg/mL of most *Rhododendron* leaf extracts did neither significantly reduce the total cell number nor affect plasma membrane integrity. However, incubation of IEC6 cells with 50 μg/mL leaf extracts of *R. polycladum, R. concinnum, R. anthopogon* ssp. *anthopogon,* and *R. hirsutum* resulted in a significant reduction in the total cell number as compared to controls (Fig. 2a). Application of the highest concentration of the majority of leaf extracts resulted in a significant decrease in the total cell number and a dramatic decrease in plasma membrane integrity (Table 2, Additional file 1: Figure S1). This suggested that the observed effects on IEC6 cell cultures were likely due to both, massive cell de-adhesion and cell death via plasma membrane rupturing of the majority of remaining cells, irrespective of the leaf extract used. Interestingly, the leaf extract of *R. ambiguum* had a remarkably divergent effect on IEC6 cells as opposed to all other extracts at 500 μg/mL: although almost all cells had lost their plasma membrane integrity, they remained adherent to the bottom of the incubation vessels (Additional file 1: Figure S1, Table 2), indicating that the extract of *R. ambiguum* potentially induces effects different from those of the other species.

**Table 2:** Total cell numbers and percentages of dead cells of IEC6 and HaCaT cell cultures which were treated with three different concentrations of *Rhododendron* leaf extracts as indicated

Treatments	Conc. μg/mL	IEC 6		HaCaT	
		Total cell numbers (Draq5™)	Dead cells (%)	Total cell numbers (Draq5™)	Dead cells (%)
*R. hippophaeoides* var. *hippophaeoides*	500	99 ± 46	81 ± 16	212 ± 57	0 ± 0
	50	494 ± 188	2 ± 0.6	283 ± 123	0.2 ± 0.2
	5	297 ± 139	1 ± 1	328 ± 138	2 ± 2
*R. minus*	500	74 ± 48	79 ± 19	221 ± 86	83 ± 12
	50	497 ± 106	0.4 ± 0.4	348 ± 113	0.4 ± 0.5
	5	683 ± 60	0.8 ± 0.2	372 ± 155	0.8 ± 0.8
*R. rubiginosum*	500	288 ± 120	97 ± 3	253 ± 122	4 ± 0.7
	50	551 ± 147	0.9 ± 1	413 ± 197	0.9 ± 0.9
	5	672 ± 101	0.5 ± 0.4	390 ± 41	0 ± 0
*R. cinnabarinum*	500	204 ± 40	100 ± 0	112 ± 23	94 ± 7
	50	475 ± 183	0.8 ± 0.8	448 ± 77	2 ± 1
	5	481 ± 128	1 ± 1	552 ± 68	2 ± 0.5
*R. ferrugineum*	500	33 ± 11	100 ± 0	490 ± 122	85 ± 15
	50	522 ± 62	1 ± 0.6	287 ± 109	1 ± 1
	5	439 ± 85	2 ± 2	481 ± 59	1 ± 1
*R. polycladum*	500	99 ± 25	100 ± 0	204 ± 62	13 ± 22
	50	274 ± 106	1 ± 1	204 ± 72	1 ± 1
	5	384 ± 104	0.9 ± 0.5	274 ± 26	0 ± 0
*R. concinnum*	500	304 ± 109	100 ± 0	143 ± 58	93 ± 6
	50	252 ± 58	45 ± 44	320 ± 66	0.5 ± 0.1
	5	586 ± 160	0.9 ± 0.8	260 ± 108	0 ± 0
*R. xanthostephanum*	500	211 ± 58	12 ± 4	127 ± 56	0 ± 0
	50	408 ± 68	0 ± 0	181 ± 70	0 ± 0
	5	460 ± 115	0.7 ± 0.6	319 ± 89	0.3 ± 0.3
*R. anthopogon* ssp. *anthopogon*	500	164 ± 41	100 ± 0	196 ± 50	90 ± 4
	50	235 ± 72	23 ± 9	370 ± 153	1 ± 1
	5	578 ± 164	2 ± 1	395 ± 157	4 ± 5
*R. ambiguum*	500	666 ± 220	99 ± 2	179 ± 60	0 ± 0
	50	439 ± 154	1 ± 1	327 ± 82	0.5 ± 0.6
	5	535 ± 119	0.7 ± 0.7	357 ± 150	0 ± 0
*R. hirsutum*	500	189 ± 65	99 ± 0.6	270 ± 113	98 ± 0.7
	50	267 ± 64	11 ± 15	258 ± 109	0.5 ± 0.1
	5	271 ± 147	1 ± 0.5	298 ± 67	0 ± 0
*R. racemosum*	500	224 ± 54	81 ± 23	123 ± 45	94 ± 3
	50	400 ± 262	0.4 ± 0.6	211 ± 33	0 ± 0
	5	471 ± 183	1 ± 0.7	225 ± 79	0 ± 0

Data are given as means ± standard deviation

HaCaT keratinocytes exposed to *Rhododendron* leaf extracts at any of the concentrations tested proved more tolerant than IEC6 cells under the same conditions. The total cell number was only significantly diminished upon incubation of HaCaT cells with 500 μg/mL leaf extracts from four *Rhododendron* species, *i.e. R. cinnabarinum*, *R.concinnum*, *R. xanthostephanum*, and *R. racemosum* (Fig. 2b, Additional file 2: Figure S2). Three out of those treatments followed the previously observed major trend: A combination of cell de-adhesion and plasma membrane disruption of HaCaT cells was observed when a high concentration of plant extract was applied (Table 2). Interestingly, the extract of *R. xanthostephanum* led to de-adhesion but not to disruption of plasma membrane integrity. Irrespective of the level of reduction in total cell number caused by 500 μg/mL of extract (Fig. 2b), five out of the 12 *Rhododendron* leaf extracts did not induce plasma membrane rupture in HaCaT cells (Table 2). This result indicated significant differences in the susceptibility of the two different cell types to the tested *Rhododendron* leaf extracts.

### Effects of Rhododendron leaf extracts on mitochondrial membrane potential

**Fig. 3:**
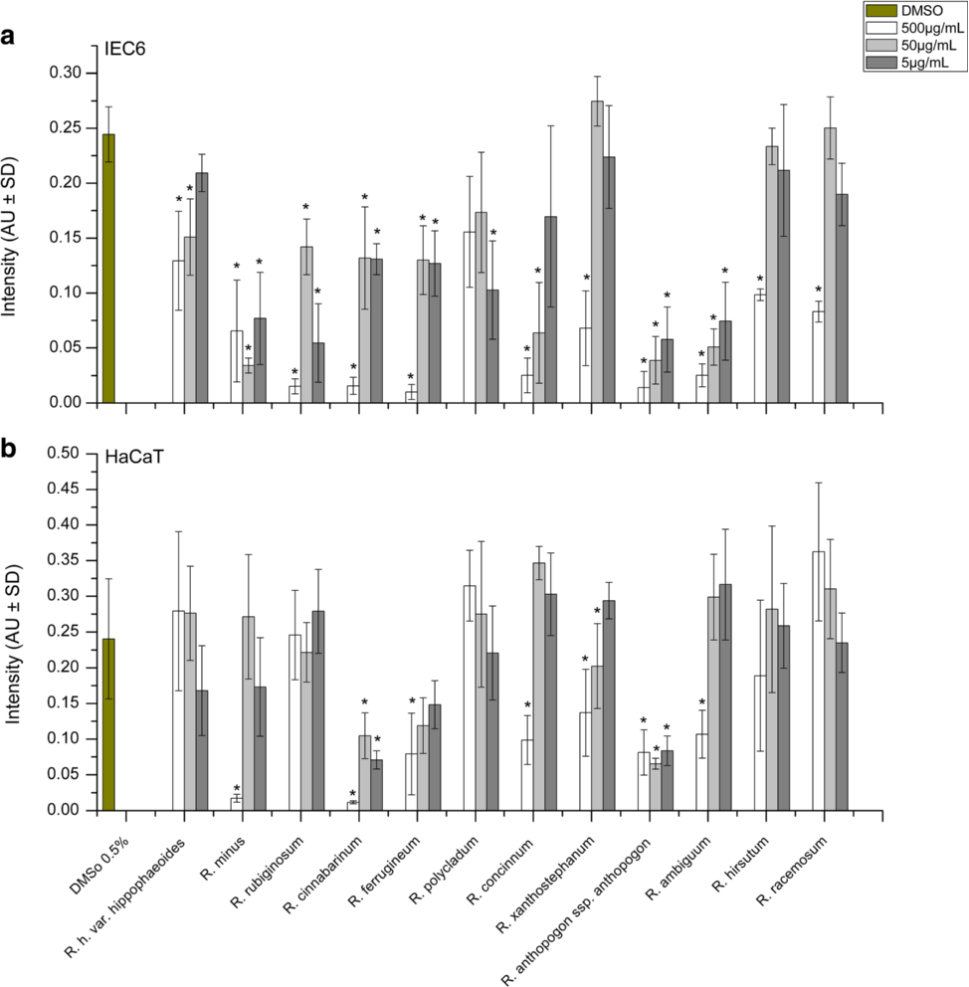
Effects of *Rhododendron* leaf extracts on the mitochondrial membrane potential of IEC6 (**a**) and HaCaT (**b**) after 24 h incubation at 37 °C with three different concentrations (5, 50, and 500 μg/mL) of leaf extract as indicated. The intensity of MitoTracker® Red CMXRos signal reflects the accumulation of the dye within the mitochondrial matrix, which depends on an intact inner mitochondrial membrane potential, and thus on the metabolic activity of the cells. Values are given as mean ± standard deviations from three independent experiments, each performed in triplicates. Statistical evaluation was performed by one way ANOVA-analysis; levels of significance are indicated as *for *p < 0.05*

**Fig. 4:**
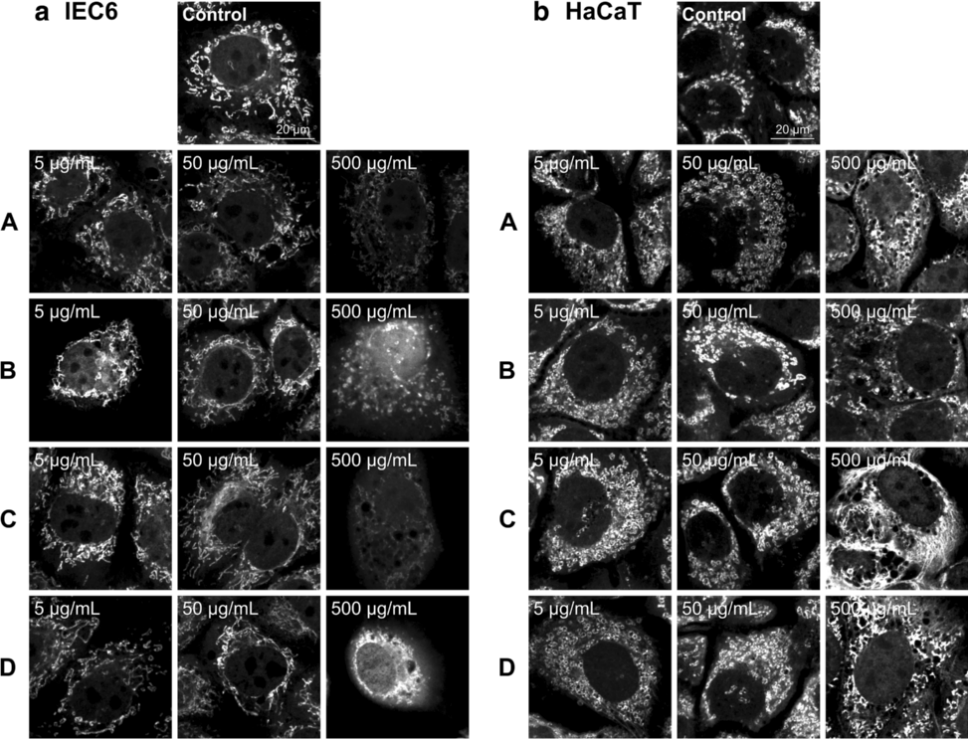
Morphological changes of mitochondria in IEC6 and HaCaT cells after 24 h exposure to three different concentrations (5, 50 and 500 μg/mL) of *Rhododendron* leaf extracts. Confocal fluorescence images of IEC6 (**a**) and HaCaT cells (**b**) labeled with MitoTracker® Red CMXRos. Cells treated with 0.5 % DMSO were used as controls. Also depicted are cells incubated with extracts from *R. hippophaeoides* var. *hippophaeoides* (*A*), *R. xanthostephanum* (*B*), *R. hirsutum* (*C*) and *R. racemosum* (*D*). Bars represent 20 μm

Changes of the mitochondrial membrane potential of IEC6 and HaCaT cells, induced by *Rhododendron* leaf extracts were determined by MitoTracker® Red CMXRos. For this, fluorescence intensity of stained mitochondria was quantified by measuring the average intensity over arbitrarily chosen inspection areas (Fig. 3). The measured staining intensity is directly proportional to the extent of metabolically active mitochondria visualized by fluorescence. In addition, *Rhododendron* leaf extract-treated and MitoTracker® Red CMXRos-stained cells were inspected under a fluorescence microscope in accordance with morphological criteria that allow for the determination of the shape of mitochondria (Fig. 4, Additional file 3: Figure S3). Typically, mitochondria of metabolically active, well-adherent IEC6 cells with an intact cytoskeleton exhibited an elongated appearance (Fig. 4a, control), while the mitochondria of HaCaT keratinocytes appeared oval or doughnut-like in shape (Fig. 4b, control).

IEC6 cells incubated with leaf extracts from all *Rhododendron* species at the highest concentration were dramatically affected with regard to the mitochondrial membrane potential as deduced from the drastically reduced MitoTracker® Red CMXRos staining although effects were somewhat milder for leaf extracts from *R. hippophaeoides* var. *hippophaeoides, R. xanthostephanum, R. hirsutum,* and *R. racemosum* (Fig. 3a). Alterations in mitochondrial structure of IEC6 cells treated with leaf extracts were frequently observed at all three concentration (Additional file 3: Figure S3). However, IEC6 cells treated with 5 or 50 μg/mL extracts from *R. hippophaeoides* var. *hippophaeoides*, *R. xanthostephanum*, *R. hirsutum,* and *R. racemosum* did not show significant differences in the metabolic activity and mitochondrial structure when compared to controls (Figs. 3a and 4a).

Effects of *Rhododendron* leaf extracts on mitochondrial structure and metabolic activity, i.e. staining intensities, were much less pronounced in HaCaT keratinocytes (Figs. 3b and 4b, Additional file 3: Figure S3b). Exceptions were observed when HaCaT cell cultures were treated with high concentrations of leaf extracts prepared from *R. minus*, *R. cinnabarinum*, *R. ferrugineum*, *R. concinnum*, *R. anthopogon* ssp. *anthopogon*, and *R. ambiguum* (Fig. 3b) with mitochondria that no longer appeared elongated but were rounded up (Fig. 4b, Additional file 3: Figure S3b).

### Analysis of the actin cytoskeleton of Rhododendron extract-treated cells

**Fig. 5:**
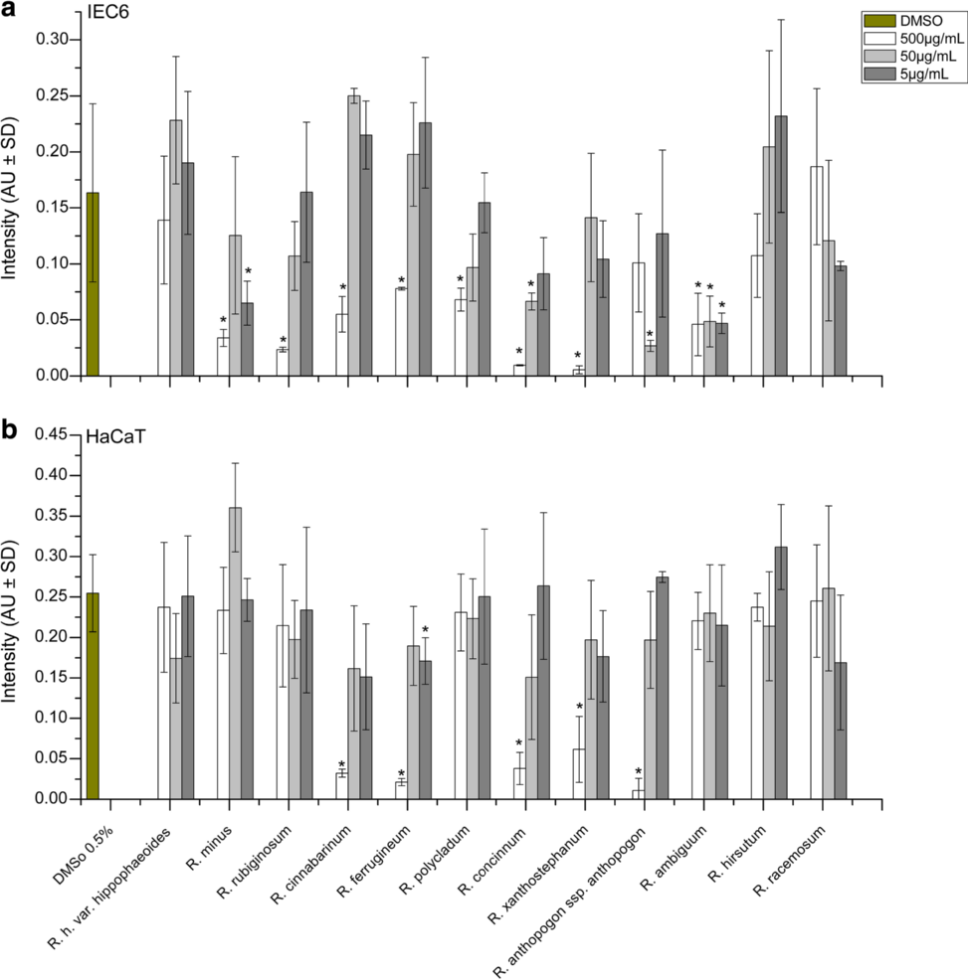
Effects of *Rhododendron* leaf extracts on the F-actin cytoskeleton of IEC6 and HaCaT cells upon incubation with three different concentrations (5, 50, and 500 μg/mL) of leaf extract for 24 h. The intensity of the phalloidin signal in IEC6 (**a**) and HaCaT cells (**b**) reflects F-actin presence, which maintains the cellular architecture. Values are given as mean ± standard deviations from three independent experiments, each performed in triplicates. Statistical evaluation was performed by one way ANOVA-analysis; levels of significance are indicated as *for *p < 0.05*

**Fig. 6:**
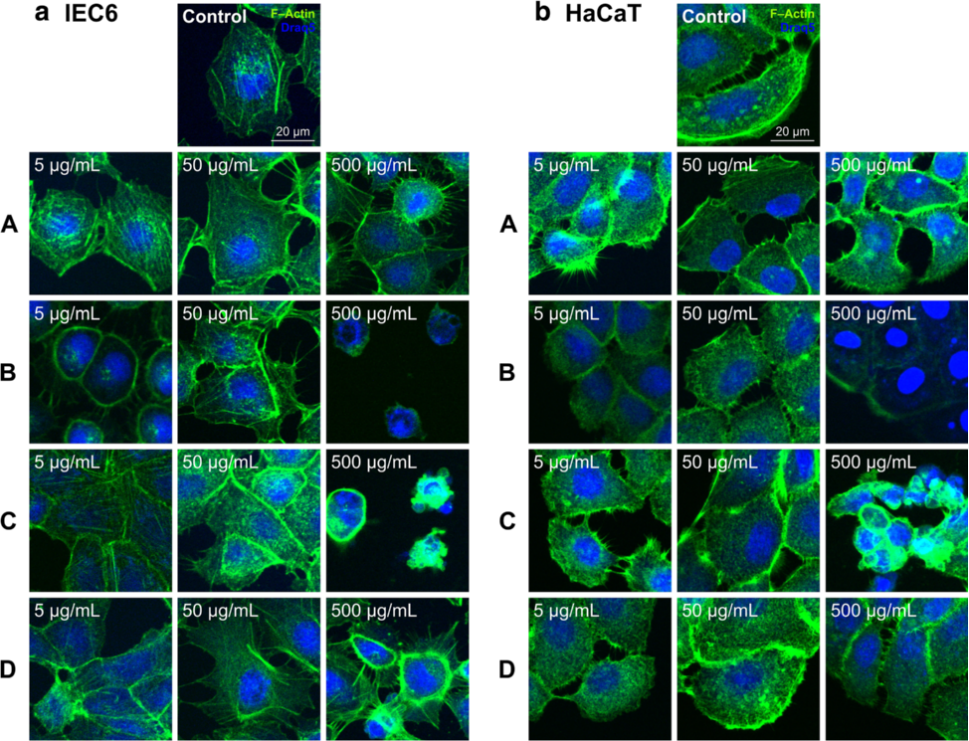
Morphological changes of F-actin structures in IEC6 and HaCaT cells after 24 h exposure to three different concentrations (5, 50 and 500 μg/mL) of *Rhododendron* leaf extracts. Confocal fluorescence images of IEC6 (**a**) and HaCaT cells (**b**) labeled with phalloidin (green) and Draq5™ (blue). Cells treated with 0.5 % DMSO served as controls for cells treated with leaf extracts of *R. hippophaeoides* var. *hippophaeoides* (*A*), *R. xanthostephanum* (*B*), *R. hirsutum* (*C*) and *R. racemosum* (*D*). Bars represent 20 μm

Next, we inspected the filamentous actin cytoskeleton of *Rhododendron* leaf extract-treated IEC6 and HaCaT cells as a measure for the preservation of the overall cellular architecture. With regard to the intensity of FITC-phalloidin staining of the F-actin system of both IEC6 and HaCaT cells, the analyses revealed mostly mild effects of the *Rhododendron* leaf extracts when applied at concentrations of 5 or 50 μg/mL (Fig. 5). Likewise, when morphologically inspecting the cytoskeleton of either cell type, no visible changes to the cortical F-actin system were caused by the lower concentrations of leaf extracts. The corresponding structures remained detectable underneath the plasma membranes of most cells (Fig. 6). Conversely, IEC6 cells exposed to any of the concentrations of *R. ambiguum* leaf extracts showed a significant decrease in the intensity of phalloidin-staining (Fig. 5a). Five of the *Rhododendron* leaf extracts applied at the highest concentration, namely *R. cinnabarinum*, *R. ferrugineum*, *R. concinnum*, *R. xanthostephanum*, and *R. anthopogon* ssp. *anthopogon*, resulted in a significantly reduced staining intensity of the filamentous actin system in both cell lines (Fig. 6). In contrast, the extracts of *R. minus*, *R. rubiginosum*, and *R. polycladum* exerted negative effects on the staining of F-actin in IEC6 cells only (Fig. 6a). This suggested a rather heterogeneous spectrum of effects on the actin cytoskeleton by various *Rhododendron* extracts. No particular morphological phenotype could be observed in association with Rhododendron extract-treated HaCaT keratinocytes (Fig. 6b, Additional file 4: Figure S4).

### Inspection of sub-cellular architecture of floating cells that detached from monolayers

The findings detailed above suggested that IEC6 and HaCaT cells remained either adherent within or to the monolayers, or that they detached upon incubation with specific *Rhododendron* leaf extracts. Such observations could be falsely interpreted as both cell types being able to tolerate exposure to cytotoxic agents only to some extent. Because the above assays were technically restricted to adherent cells in monolayers, we next analyzed the fraction of free-floating cells which detached during treatment with *Rhododendron* leaf extracts using the same staining methods as described above. Therefore, Draq5™ staining additionally served to examine the status of nuclear DNA and to identify morphological alterations of the nuclei, such as those that are typical for cells undergoing programmed cell death.

**Fig. 7:**
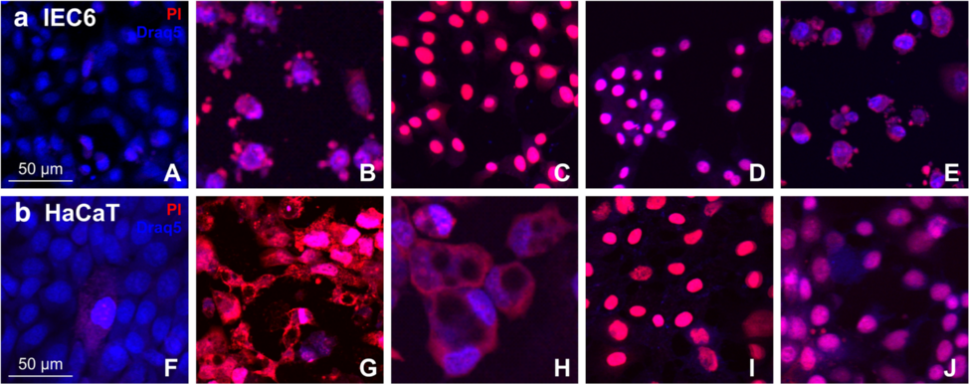
Plasma membrane integrity and cell death by apoptosis as induced by a 24 h-exposure of IEC6 cells and HaCaT keratinocytes to 500 μg/mL of specific *Rhododendron* leaf extracts. Merged micrographs taken with a confocal laser scanning microscope depict IEC6 (**a**; *panels A-E*) and HaCaT cells (**b**; *panels F-J*). Violet signals in merged images are due to overlapping red, PI-derived signals, in cells with ruptured plasma membranes, and blue, Draq5™ staining of nuclei in all cells. Pictures A and F are control cells treated with 0.5 % DMSO, while cells in all other panels were incubated with extracts from *R. cinnabarinum* (*B and G*), *R. ferrugineum* (*C and H*), *R. minus* (*D and I*) and *R. hippophaeoides* var. *hippophaeoides* (*E and J*). Bars represent 50 μm

Treatment of IEC6 cells with 500 μg/mL leaf extracts from *R. cinnabarinum* and subsequent analysis of the detached cells revealed nuclear condensation, cell shrinkage, rounding-up, loss of contacts with adjacent cells, formation of typical membrane blebs and occurrence of apoptotic bodies (Fig. 7a). These results suggested that leaf extracts of this particular plant species may induce apoptosis.

However, IEC6 cells exposed to 500 μg/mL *R. ferrugineum* leaf extracts became pycnotic and the actin filaments formed a ring surrounding the nucleus. In addition, IEC6 cells treated with leaf extracts from *R. minus*, *R. rubiginosum,* and *R. ambiguum* showed different stages of chromatin condensation and shrinkage of the nuclei (Fig. 7). Thus, five *Rhododendron* leaf extracts, namely *R. hippophaeoides* var. *hippophaeoides*, *R. cinnabarinum*, *R. ferrugineum*, *R. xanthostephanum*, and *R. racemosum* induced signs closely related to the classical symptoms of programmed cell death –apoptosis– where the treated cells also exhibited typical phenotypes like formation of plasma membrane blebs.

HaCaT cells too showed cellular changes indicative of cell death upon exposure to 500 μg/mL *Rhododendron* leaf extracts. However, these were different from the phenotypes observed in treated IEC6 cell cultures. HaCaT cells treated with leaf extracts from either *R. cinnabarinum* or *R. ferrugineum* displayed signs of the final stages of cell death, reminiscent of cornification, because they exhibited intense PI staining throughout the nuclei and the cytoplasm, while some cells had lost their nuclei altogether (Fig. 7b, G and H).

Moreover, exposure of HaCaT cells to *R. minus*, *R. concinnum*, and *R. anthopogon* ssp. *anthopogon* induced changes that featured shrinkage of nuclei and chromatin condensation (Fig. 7b, I).

### Investigation of apoptotic cell death pathways through determination of procaspase-3 activation

**Fig. 8:**
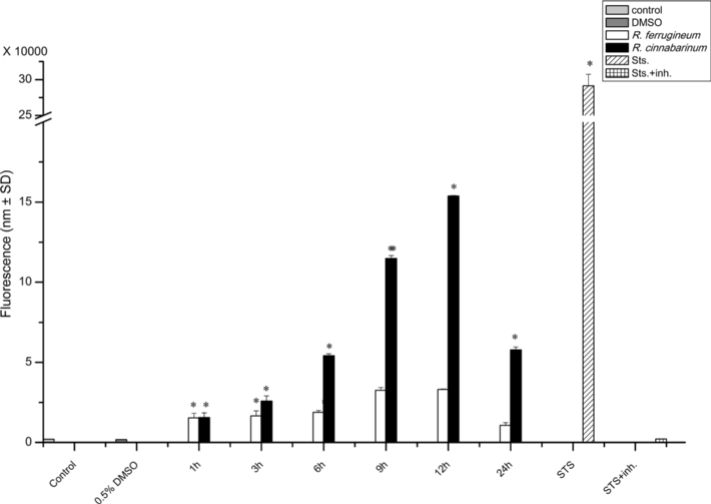
Detection of caspase-3 activity in IEC6 cells. Cells were treated with 500 μg/mL of leaf extracts from *R. cinnabarinum* (**a**) and *R. ferrugineum* (**b**) for the indicated time intervals. Reactions were carried out at room temperature and fluorescence was measured in a fluorescence microplate reader using 496 nm for excitation and emission was detected at 520 nm. Non-treated cells and cells treated with DMSO (0.5 %) were used as negative controls, while staurosporine (10 μg/mL) treatment was used as a positive control (apoptosis inducer). Values are given as mean ± standard deviations from three independent experiments, each performed in triplicates. Statistical evaluation was performed by one way ANOVA-analysis; levels of significance are indicated as *for *p < 0.05*

In order to substantiate the interpretation of some of the observed morphological changes induced by specific *Rhododendron* leaf extracts, the cells grown in monolayers and treated with the extracts were subjected to an additional apoptosis-proving assay. Besides some of the above noted symptoms, one definitive characteristic of apoptosis is the activation of procaspase-3. Therefore, a caspase-3 activity assay was applied to all treatments of IEC6 cells. Incubation of the cells with 500 μg/mL leaf extracts from any of the 12 *Rhododendron* species indeed induced apoptosis as evidenced by a significant increase in the levels of caspase-3 activity (data not shown). There was no significant activation of procaspase-3 when IEC6 cells were treated with 5 or 50 μg/mL of all leaf extracts prepared from *Rhododendron* species. Interestingly, cells treated with extracts from *R. cinnabarinum* and *R. ferrugineum* at concentrations of 500 μg/mL clearly exhibited phenotypic changes characteristic to apoptosis (Fig. 7). Thus, we selected the leaf extracts of these two *Rhododendron* species to analyze procaspase-3 activation in free-floating IEC6 cells in a time-dependent manner (Fig. 8). The results revealed a steady increase in caspase-3 activity levels until 12 h of treatment with extracts of *R. cinnabarinum,* and to a five-fold lesser extent upon treatment with *R. ferrugineum* extracts. However, caspase-3 activity decreased during the next 12 h. These results argue that components contained in *Rhododendron* leaf extracts induce apoptosis in IEC6 cells when applied in high enough concentrations.

### Summarizing integration of the results achieved with a variety of cell toxicity assays

**Fig. 9:**
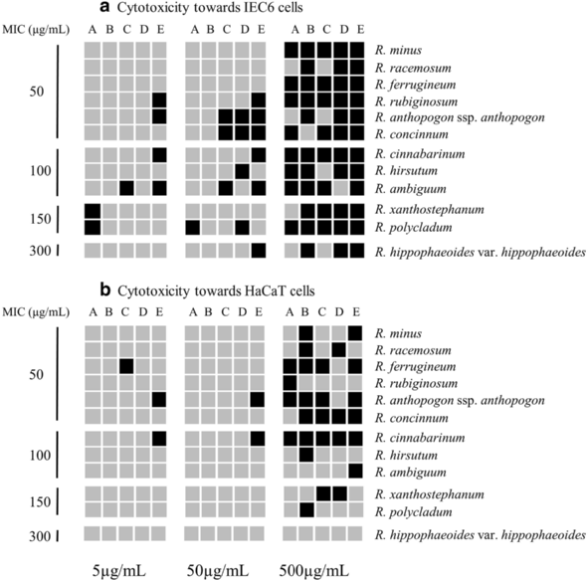
Profile map summarizing the results of cell biological assessment assays, combining the effects of *Rhododendron* crude extracts against *B. subtilis* depicted by minimum inhibitory concentration. The twelve *Rhododendron* species were classified into four groups (i.e. 50, 100, 150, and 300 μg/mL) according to the MIC results (black vertical line). Three concentrations (5, 50, and 500 μg/mL) of *Rhododendron* crude extracts were applied to the two different cell lines for 24 h representing low, medium, and high antimicrobial activity, respectively. The grey shading represents the toxicity that *Rhododendron* crude extracts exerted on mammalian cells, i.e. non-toxic extracts are depicted in light grey, and cytotoxic extracts are shown by dark grey boxes. Panel (**a**) represents the results of IEC6 cells for the assays on cell viability and proliferation rates (*A*), plasma membrane integrity (*B*), cellular architecture *(C)*, total cell numbers *(D)*, and cellular metabolism *(E)*. Panel (**b**) represents the corresponding results for HaCaT cells

The partially complex data acquired herein with different cell toxicity analysis assays are summarized by grouping the 12 *Rhododendron* species according to their antibacterial effectiveness with respective MICs of 50, 100, 150, or 300 μg/ml, and qualitatively comparing their effects against both cell types (Fig. 9). In general, most *Rhododendron* leaf extracts exerted more pronounced effects on IEC6 intestine epithelial cells as compared to HaCaT keratinocytes, when applied at high concentrations (500 μg/mL). *R. hippophaeoides* var. *hippophaeoides*, which exhibited the lowest antibacterial effect, also proved to be least toxic towards both mammalian cell types. A total of five *Rhododendron* extracts with high antibacterial potential (MIC of 50 μg/mL) did not reveal cytotoxicity against the mammalian cell lines in any of the tested assays when applied at 50 μg/mL, indicating that these extracts are unlikely to harm mammalian cells while killing bacterial cells. Thus, these five extracts are the candidates to be further assessed for possibly containing bio-active compounds with antimicrobial potencies, while still proving safe to be applied onto epidermal or intestine mucosal cell monolayers. The corresponding plant species were *R. minus*, *R. racemosum*, *R. ferrugineum*, *R. rubiginosum*, and *R. concinnum*. Interestingly, only the extracts of *R. minus* and *R. racemosum* proved to be non-cytotoxic to both intestine epithelial cells and keratinocytes (Fig. 9), suggesting they are the most promising candidates for future investigations on the search for optimized antibiotics in bio-active plant extract and, therefore, to be used for the identification and purification of specific compounds derived from *Rhododendron*.

## Discussion

To date, there are only few medicinal formulations on the market that contain compounds derived from *Rhododendron.* These comprise ‘Rhomitoxin’ used to treat hypertension, and ‘*Rhododendron* cp paste’ used to relieve pain in arthritis [10]. In addition, only few *in vitro* and *in vivo* studies with specific *Rhododendron* extracts and compounds isolated thereof have been reported that validated plant extracts as being useful in traditional remedies [20]. Importantly, plants of the genus *Rhododendron* are more commonly used as alternative medicine in the geographic regions of their natural habitats, i.e., Nepal, Northeastern India, Western and Central China, or Indonesia [20]. This may be due to the fact that the precise chemical composition of medicinal formulations is often not very well defined [40, 41]. However, *Rhododendron* plants are known to synthesize a large number of chemical compounds, some of which exhibit attested pharmacological activities [42–45]. Several of these chemical compounds have been identified to belong to the pro-anthocyanidins, polyphenols, or terpenoids which are typically synthesized by plants reacting in defense to pathogenic infection or inflictions caused by herbivores [46, 47].

Not surprisingly, various plant-derived compounds exert severe cytotoxic or mutagenic effects when applied to animal cells and tissues [48, 49]. Intoxication of domesticated or wild animals feeding on *Rhododendron* plants have been repeatedly reported and were linked to the presence of grayano-toxins [50–52]. Therefore, a comprehensive number of cytotoxicity studies involving mammalian cells or tissue cultures must be conducted before a given extract or a defined Rhododendron-derived compound can eventually be considered for testing on animal models, or even enter clinical trials [53, 54].

To the best of our knowledge, none of the previous studies had comprehensively analyzed the cytotoxicity of a group of pharmaceutically interesting *Rhododendron* species. Consequently, the current study introduces a multi-facetted approach, consisting of five different cytotoxicity assays, in order to investigate the effects of *Rhododendron* leaf extracts on cellular structure, metabolic activity, and viability of two different types of mammalian cells.

The results obtained herein show that treating IEC6 and HaCaT cells with low concentrations of leaf extracts prepared from any of the 12 *Rhododendron* species exhibited rather mild or no cytotoxic effects, whereas the use of high concentrations (500 μg/mL) resulted in a rather expected and remarkable cytotoxicity. A total of five *Rhododendron* species exhibited high antibacterial activities with MICs of 50 μg/mL and proved to be non-cytotoxic at this concentration. Interestingly, extracts of *R. minus* and *R. racemosum* were non-toxic to either cell lines, which makes them promising candidates for future studies. In contrast, incubation of either of the two cell lines with 500 μg/mL of the other *Rhododendron* leaf extracts resulted in severe structural and functional alterations often associated with signs of apoptosis. Our study thus confirmed that simultaneous analysis of several, albeit partially unlinked or only indirectly linked cellular parameters, is a convenient tool to separate potentially cytotoxic extracts from their ‘safe-to-use’ *Rhododendron* extracts counterparts, thus overcoming technical short-comings of previous studies aiming at high-throughput screening.

Our results demonstrated that the incubation of cells with high concentrations of *Rhododendron* leaf extracts induced apoptosis specifically in intestine epithelial cells. Interestingly, only two extracts, namely those of *R. cinnabarinum* and *R. ferrugineum*, shared a similar pattern of cytotoxicity in all assays tested in this study. Leaf extracts of these two *Rhododendron* species were capable of inducing procaspase-3 activation prominently in IEC6 cells. The results of this study concur with other studies that have shown several secondary metabolic compounds from *Rhododendron* species to induce apoptosis in cultures of different mammalian cell lines [55, 57].

Overall, keratinocytes were more resistant to cytotoxicity exerted upon incubation with *Rhododendron* leaf extracts than IEC6 cells. Resistance of HaCaT cells against cytotoxic agents was observed by us previously when studying dust exposure [32]. This remarkable feature of keratinocytes might be due to the specific lipid composition of their membranes and their ability to build a stratified epithelium when exposed to air during cornification [32, 57, 58].

## Conclusion

Using a comprehensive approach, the cytotoxicity of those *Rhododendron* species that had previously been shown to exhibit the highest antibacterial activities was determined. As such, we managed to continue our ongoing approach in identifying pharmaceutically feasible antibiotics or lead structures. Utilizing two tester cell lines as relevant models for the envisioned ectopic or oral treatment and applying several different cell biological assays, proved to be a suitable combination of screening tools. Two out of the 12 *Rhododendron* species with antibacterial properties exhibit the desired traits: the extracts of *R. minus* and *R. racemosum* were both non-cytotoxic at a concentration at where they efficiently produced an inhibition zone for *B. subtilis*.

Furthermore, we could conclude that *Rhododendron* leaf extracts induced apoptosis, as evidenced by typical alterations of the cellular phenotypes (chromatin condensation and formation of plasma membrane blebs) as well as by the increasing levels of active caspase-3 when cells were exposed to higher extract concentrations. In the future, we will extend our current study in order to determine whether the specific apoptosis-inducing effects of *R. cinnabarinum* and *R. ferrugineum* can be used to selectively target cancer cells, such as colorectal carcinoma cells.

In our future research, we will focus on phyto-chemically identifying the actual active compounds present in the leaf extracts derived from different *Rhododendron* species. We plan to determine the IC 50 values and to study their potential cytotoxic effects through a repertoire of different methods similar to the cell biological screening tool box laid out in the current study.

## Additional files

Additional file 1: Figure S1. Overview of plasma membrane integrity and apoptotic cell death induced by 24h exposure of IEC6 cells to three different concentrations (5, 50, and 500 μg/mL) of *Rhododendron* leaf extracts. Single channel fluorescence, phase contrast and merged micrographs taken with a confocal laser scanning microscope. Violet signals in merged pictures are due to overlapping red, PI-derived signals with blue Draq5™ staining of the nuclei. Cells treated with 0.5 % DMSO served as controls, A) *R. hippophaeoides* var. *hippophaeoides,* B) *R. minus,* C) *R. rubiginosum,* D) *R. cinnabarinum,* E) *R. ferrugineum,* F) *R. polycladum,* G) *R. concinnum,* H) *R. xanthostephanum,* I) *R. anthopogon* ssp. *anthopogon,* J) *R. ambiguum,* K) *R. hirsutum,* and L) *R. racemosum.* Bar represents 250 μm. (TIFF 6203 kb) Additional file 2: Figure S2. Overview of plasma membrane integrity and apoptotic cell death induced by 24h exposure of HaCaT keratinocytes to three different concentrations (5, 50, and 500 μg/mL) of *Rhododendron* leaf extracts. Single channel fluorescence, phase contrast and merged micrographs taken with a confocal laser scanning microscope. Violet signals in merged pictures are due to overlapping red, PI-derived signals with blue Draq5™ staining of the nuclei. Cells treated with 0.5 % DMSO served as controls, A) *R. hippophaeoides* var. *hippophaeoides,* B) *R. minus,* C) *R. rubiginosum,* D) *R. cinnabarinum,* E) *R. ferrugineum,* F) *R. polycladum,* G) *R. concinnum,* H) *R. xanthostephanum,* I) *R. anthopogon* ssp. *anthopogon,* J) *R. ambiguum,* K) *R. hirsutum,* and L) *R. racemosum.* Bar represents 250 μm. (TIFF 6203 kb) Additional file 3: Figure S3. Overview of mitochondrial morphology in IEC6 and HaCaT cells after a 24h-exposure to three different concentrations (5, 50 and 500 μg/mL) of *Rhododendron* leaf extracts. Confocal fluorescence images of IEC6 (a, left) and HaCaT (b, right) cells labeled with MitoTracker® Red CMXRos. Cells treated with 0.5 % DMSO served as controls, A) *R. hippophaeoides* var. *hippophaeoides,* B) *R. minus,* C) *R. rubiginosum,* D) *R. cinnabarinum,* E) *R. ferrugineum,* F) *R. polycladum,* G) *R. concinnum,* H) *R. xanthostephanum,* I) *R. anthopogon* ssp. *anthopogon,* J) *R. ambiguum,* K) *R. hirsutum,* and L) *R. racemosum.* Bars represent 50 μm. (TIFF 11640 kb) Additional file 4: Figure S4. Overview of the structure of the F-actin system in IEC6 and HaCaT cells after a 24h-exposure to three different concentrations (5, 50 and 500 μg/mL) of *Rhododendron* leaf extracts. Confocal fluorescence images of IEC6 (a, left) and HaCaT (b, right) labeled with phalloidin (green) and Draq5™ (blue). Cells treated with 0.5 % DMSO served as controls, A) *R. hippophaeoides* var. *hippophaeoides,* B) *R. minus,* C) *R. rubiginosum,* D) *R. cinnabarinum,* E) *R. ferrugineum,* F) *R. polycladum,* G) *R. concinnum,* H) *R. xanthostephanum,* I) *R. anthopogon* ssp. *anthopogon,* J) *R. ambiguum,* K) *R. hirsutum,* and L) *R. racemosum.* Bars represent 50 μm. (TIFF 8731 kb)

## References

[1] Semkina OA (2005). Ointments, gels, liniments, and creams containing phytopreparations (a review). Pharm Chem J.

[2] Fennell CW, Lindsey KL, McGaw LJ, Sparg SG, Stafford GI, Elgorashi EE, et al. Assessing African medicinal plants for efficacy and safety: pharmacological screening and toxicology. J Ethnopharmacol. 2004;94(2–3):205–17. 10.1016/j.jep.2004.05.012 15325724

[3] Solecki RS (1975). Shanidar IV, a neanderthal flower burial in Northern Iraq. Science.

[4] Zhang X (2002). WHO traditional medicine strategy 2002–2005.

[5] Farnsworth NR, D. Soejarto. Global importance of medicinal plants. The conservation of medicinal plants 1991:p. 25–51.

[6] Kim H-S (2005). Do not put too much value on conventional medicines. J Ethnopharmacol.

[7] Cragg GM, Newman DJ (2005). Plants as a source of anti-cancer agents. J Ethnopharmacol.

[8] Chicca A, Pellati F, Adinolfi B, Matthias A, Massarelli I, Benvenuti S, et al. Cytotoxic activity of polyacetylenes and polyenes isolated from roots of Echinacea pallida. Br J Pharmacol. 2008;153(5):879–85. 10.1038/sj.bjp.0707639 PMC2267283 18193076

[9] De Silva T (1997). Industrial utilization of medicinal plants in developing countries.

[10] Fabricant DS, Farnsworth NR (2001). The value of plants used in traditional medicine for drug discovery. Environ Health Perspect.

[11] Chichilnisky, G. Property rights on biodiversity and the pharmaceutical industry. Available at SSRN 1374617 1993.

[12] King SR, Carlson TJ, Moran K (1996). Biological diversity, indigenous knowledge, drug discovery and intellectual property rights: creating reciprocity and maintaining relationships. J Ethnopharmacol.

[13] Kartal M (2007). Intellectual property protection in the natural product drug discovery, traditional herbal medicine and herbal medicinal products. Phytother Res.

[14] Cronquist A. The evolution and classification of flowering plants. Bronx, New York: New York Botanical Garden; 1968.

[15] Palombo EA. Traditional medicinal plant extracts and natural products with activity against oral bacteria: potential application in the prevention and treatment of oral diseases. Evidence-Based Complementary and Alternative Medicine. 2011;2011:680354. 10.1093/ecam/nep067 PMC3145422 19596745

[16] Yassin GH, Koek JH, Jayaraman S, Kuhnert N. Identification of novel homologous series of polyhydroxylated theasinensins and theanaphthoquinones in the SII fraction of black tea thearubigins using ESI/HPLC tandem mass spectrometry. J Agric Food Chem. 2014;62(40):9848–59. 10.1021/jf502220c 25263270

[17] Elliott M, Liu Y (2010). The nine rights of medication administration: an overview. Br J Nurs.

[18] Tam TW, Liu R, Saleem A, Arnason JT, Krantis A, Haddad PS. The effect of Cree traditional medicinal teas on the activity of human cytochrome P450-mediated metabolism. J Ethnopharmacol. 2014;155(1):841–6. 10.1016/j.jep.2014.06.045 24971793

[19] Tiwari ON, Chauhan UK (2005). Genus Rhododendron status in Sikkim Himalaya: an assessment. J Am Rhododendron Soc.

[20] Popescu R, Kopp B (2013). The genus Rhododendron: an ethnopharmacological and toxicological review. J Ethnopharmacol.

[21] Shakeel-U-Rehman, Khan R, Bhat KA, Raja AF, Shawl AS, Alam MS. Isolation, characterisation and antibacterial activity studies of coumarins from Rhododendron lepidotum Wall. ex G. Don, Ericaceae*.* Revista Brasileira de Farmacognosia, 2010. 20:886–90.

[22] Baral B, Vaidya GS, Maharjan BL, Da Silva JAT. Phytochemical and antimicrobial characterization of rhododendron anthopogon from high Nepalese Himalaya. Botanica Lithuanica. 2015;20(2):142–52.

[23] Kupeli E, Tatli II, Akdemir ZS, Yesilada E. Bioassay-guided isolation of anti-inflammatory and antinociceptive glycoterpenoids from the flowers of Verbascum lasianthum Boiss. ex Bentham. J Ethnopharmacol. 2007;110(3):444–50. 10.1016/j.jep.2006.10.004 17123759

[24] Nisar M, Ali S, Muhammed N, Gillani SN, Shah MR, Khan H, et al. Antinociceptive and anti-inflammatory potential of Rhododendron arboreum bark. Toxicol Ind Health. 2014. Epub ahead of print. 10.1177/0748233714555391 25501256

[25] Rezk A, Nolzen J, Schepker H, Albach DC, Brix K, Ullrich MS. Phylogenetic spectrum and analysis of antibacterial activities of leaf extracts from plants of the genus Rhododendron. BMC Complement Altern Med. 2015;15(1):1–10. 10.1186/s12906-015-0596-5 PMC4367927 25879877

[26] Seephonkai P, Popescu R, Zehl M, Krupitza G, Urban E, Kopp B. Ferruginenes a − C from Rhododendron ferrugineum and their cytotoxic evaluation. J Nat Prod. 2011;74(4):712–7. 10.1021/np100778k 21443171

[27] Li X, Jin H, Chen G, Yan S, Shen Y, Yang M, et al. Study advances on chemical constituents and pharmacological activities of the Tibetan medicine Dali. Nat Product Res & Development. 2008;20(6):1125–8.

[28] Niles AL, Moravec RA, Riss TL (2009). In vitro viability and cytotoxicity testing and same-well multi-parametric combinations for high throughput screening. Current Chemical Genomics.

[29] Thomas C, Oates PS (2002). IEC-6 cells are an appropriate model of intestinal iron absorption in rats. J Nutr.

[30] Mayer K, Vreeman A, Qu H, Brix K. Release of endo-lysosomal cathepsins B, D, and L from IEC6 cells in a cell culture model mimicking intestinal manipulation. Biol Chem. 2009;390(5/6):471–80. 10.1515/BC.2009.047 19284293

[31] Buth H, Luigi Buttigieg P, Ostafe R, Rehders M, Dannenmann SR, Schaschke N, et al. Cathepsin B is essential for regeneration of scratch-wounded normal human epidermal keratinocytes. Eur J Cell Biol. 2007;86(11–12):747–61. 10.1016/j.ejcb.2007.03.009 17651862

[32] Rehders M, Grosshäuser BB, Smarandache A, Sadhukhan A, Mirastschijski U, Kempf J, et al. Effects of lunar and mars dust simulants on HaCaT keratinocytes and CHO-K1 fibroblasts. Adv Space Res. 2011;47(7):1200–13.

[33] Huet O, Petit JM, Ratinaud MH, Julien R. NADH-dependent dehydrogenase activity estimation by flow cytometric analysis of 3-(4,5-dimethylthiazolyl-2-yl)-2,5-diphenyltetrazolium bromide (MTT) reduction. Cytometry. 1992;13(5):532–9. 10.1002/cyto.990130513 1633732

[34] Mosmann T (1983). Rapid colorimetric assay for cellular growth and survival: application to proliferation and cytotoxicity assays. J Immunol Methods.

[35] Twentyman PR, Luscombe M (1987). A study of some variables in a tetrazolium dye (MTT) based assay for cell growth and chemosensitivity. Br J Cancer.

[36] Arampatzidou M, Rehders M, Dauth S, Yu DM, Tedelind S, Brix K. Imaging of protease functions-current guide to spotting cysteine cathepsins in classical and novel scenes of action in mammalian epithelial cells and tissues. Ital J Anat Embryol. 2011;116(1):1–19. 21898969

[37] Burton K (1956). A study of the conditions and mechanism of the diphenylamine reaction for the colorimetric estimation of deoxyribonucleic acid. Biochem J.

[38] Cockerill FR, Wikler MA, Adler J, Dudley MN, Eliopoulos GM, Ferraro MJ, et al. Methods for Dilution Antimicrobial Susceptibility Tests for Bacteria that grow Aerobically. Approved Standard - Ninth Edition. CLSI document M07-A9. Wayne, PA: Clinical and Laboratory Standard Institute. 2012.

[39] Carpenter A, Jones TR, Lamprecht MR, Clarke C, Kang IH, Friman O. Cell Profiler: image analysis software for identifying and quantifying cell phenotypes. Genome Biol. 2006;7(10):R100. 10.1186/gb-2006-7-10-r100 PMC1794559 17076895

[40] Iwata N, Wang N, Yao X, Kitanaka S. Structures and histamine release inhibitory effects of prenylated orcinol derivatives from rhododendron dauricum1. J Nat Prod. 2004;67(7):1106–9. 10.1021/np0303916 15270561

[41] Bhattacharyya D (2011). Rhododendron species and their uses with special reference to Himalayas– a review. Assam University J Sci & Technology: Biological and Environ Sci.

[42] Peng YY, Liu FH, Ye JN (2004). Determination of bioactive flavonoids in *Rhododendron* dauricum L. By capillary electrophoresis with electrochemical detection. Chromatographia.

[43] Cao Y, Chu Q, Ye J (2004). Chromatographic and electrophoretic methods for pharmaceutically active compounds in Rhododendron dauricum. J Chromatogr B.

[44] Zou H-Y, Luo J, Xu DR, Kong LY. Tandem Solid-Phase Extraction Followed by HPLC–ESI/QTOF/MS/MS for Rapid Screening and Structural Identification of Trace Diterpenoids in Flowers of Rhododendron molle. Phytochem Anal. 2014;25(3):255–65. 10.1002/pca.2501 24453183

[45] Qiang Y, Zhou B, Gao K (2011). Chemical constituents of plants from the genus Rhododendron. Chem Biodivers.

[46] Amil-Ruiz F, Bianco-Portales R, Muñoz-Blanco J, Caballero JL. The strawberry plant defense mechanism: a molecular review. Plant Cell Physiol. 2011;52(11):1873–903. 10.1093/pcp/pcr136 21984602

[47] Jaiswal R, Jayasinghe L, Kuhnert N (2012). Identification and characterization of proanthocyanidins of 16 members of the Rhododendron genus (Ericaceae) by tandem LC–MS. J Mass Spectrom.

[48] Santos FV, Colus IM, Silva MA, Vilegas W, Varanda EA. Assessment of DNA damage by extracts and fractions of Strychnos pseudoquina, a Brazilian medicinal plant with antiulcerogenic activity. Food Chem Toxicol. 2006;44(9):1585–9. 10.1016/j.fct.2006.03.012 16730111

[49] Mei N, Heflich RH, Chou MW, Chen T. Mutations induced by the carcinogenic pyrrolizidine alkaloid riddelliine in the liver cII gene of transgenic Big blue rats. Chem Res Toxicol. 2004;17(6):814–8. 10.1021/tx049955b PMC6375673 15206902

[50] Farias Vargas Junior S, Marcolongo-Pereira C, Halinski-Silveira D, Grecco FB, Raffi MB, Schild AL, et al. Rhododendron simsii poisoning in goats in Southern Brazil. Ciência Rural. 2014;44(7):1249–52.

[51] Puschner B, Holstege DM, Lamberski N. Grayanotoxin poisoning in three goats. J Am Vet Med Assoc. 2001;218(4):573–5. 527–78. 10.2460/javma.2001.218.573 11229512

[52] Berny P, Caloni F, Croubels S, Sachana M, Vandenbroucke V, Davanzo F, et al. Animal poisoning in Europe. Part 2: companion animals. Vet J. 2010;183(3):255–9. 10.1016/j.tvjl.2009.03.034 19553146

[53] Ernst E (2004). Systematic reviews of herbal medicines. Am J Med.

[54] Rietjens IMCM, Boersma MG, van der Woude H, Jeurissen SMF, Schutte ME, Alink GM. Flavonoids and alkenylbenzenes: mechanisms of mutagenic action and carcinogenic risk. Mutation research/fundamental and molecular mechanisms of mutagenesis. Genes and Environment Bridging the Gap. 2005;574(1–2):124–38. 10.1016/j.mrfmmm.2005.01.028 15914212

[55] Li FR, Yu FX, Yao ST, Si YH, Zhang W, Gao LL. Hyperin extracted from Manchurian rhododendron leaf induces apoptosis in human endometrial cancer cells through a mitochondrial pathway. Asian Pac J Cancer Prev. 2012;13(8):3653–6. 10.7314/apjcp.2012.13.8.3653 23098449

[56] Way TD, Tsai SJ, Wang CM, Ho CT, Chou CH. Chemical constituents of Rhododendron formosanum show pronounced growth inhibitory effect on non-small-cell lung carcinoma cells. J Agric Food Chem. 2014;62(4):875–84. 10.1021/jf404243p 24447325

[57] Eckert RL, Rorke EA (1989). Molecular biology of keratinocyte differentiation. Environ Health Perspect.

[58] Yuan J, Kroemer G (2010). Alternative cell death mechanisms in development and beyond. Genes Dev.

