# Supplementary figures and images for: Assessment of cytotoxicity exerted by leaf extracts from plants of the genus Rhododendron towards epidermal keratinocytes and intestine epithelial cells
Source: BMC Complement Altern Med. 2015 Oct 15;15:364. doi: 10.1186/s12906-015-0860-8 (PMC4608053; doi:10.1186/s12906-015-0860-8)

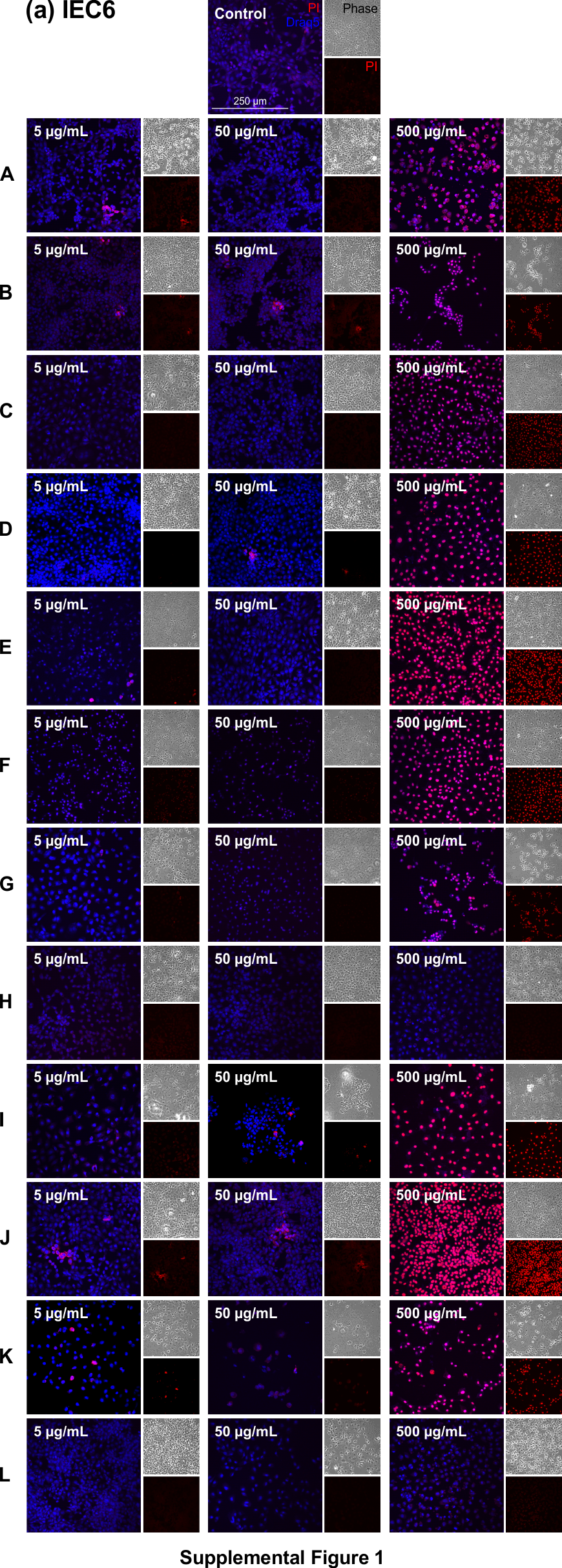

Supplement: Additional file 1: Figure S1. — Overview of plasma membrane integrity and apoptotic cell death induced by 24h exposure of IEC6 cells to three different concentrations (5, 50, and 500 μg/mL) of Rhododendron leaf extracts. Single channel fluorescence, phase contrast and merged micrographs taken with a confocal laser scanning microscope. Violet signals in merged pictures are due to overlapping red, PI-derived signals with blue Draq5™ staining of the nuclei. Cells treated with 0.5 % DMSO served as controls, A) R. hippophaeoides var. hippophaeoides, B) R. minus, C) R. rubiginosum, D) R. cinnabarinum, E) R. ferrugineum, F) R. polycladum, G) R. concinnum, H) R. xanthostephanum, I) R. anthopogon ssp. anthopogon, J) R. ambiguum, K) R. hirsutum, and L) R. racemosum. Bar represents 250 μm. (TIFF 6203 kb) [file 12906_2015_860_MOESM1_ESM.tif]

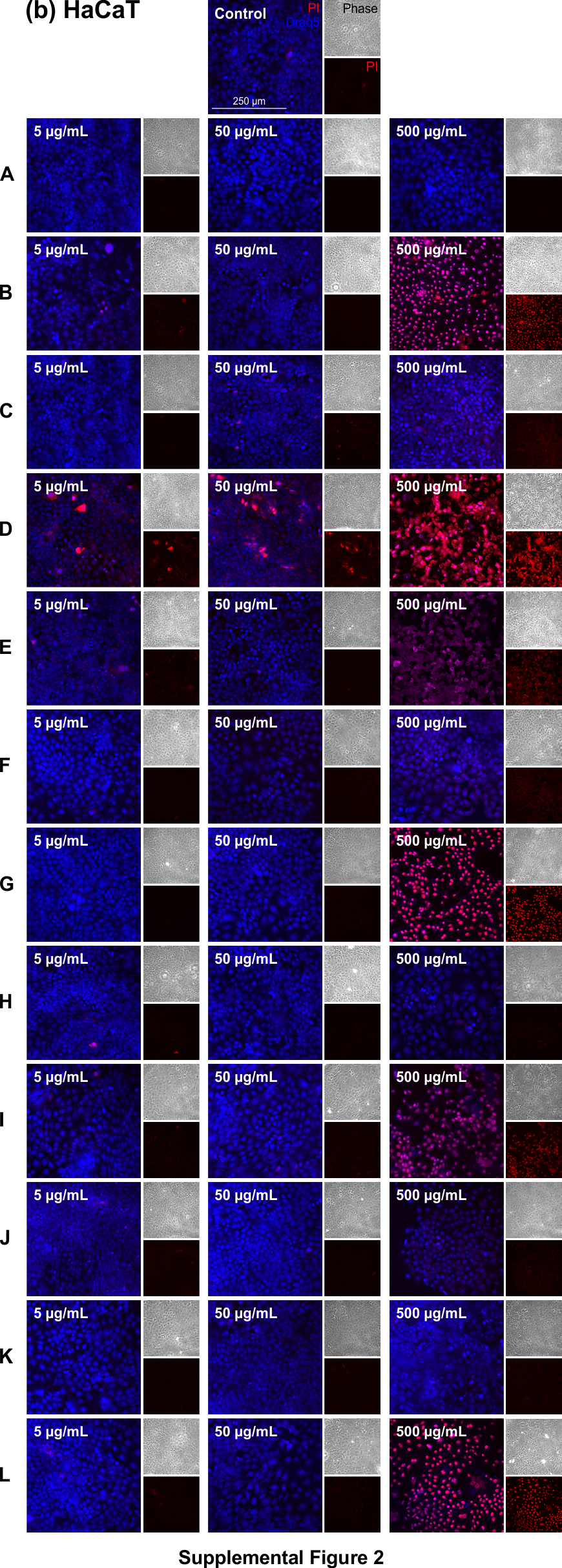

Supplement: Additional file 2: Figure S2. — Overview of plasma membrane integrity and apoptotic cell death induced by 24h exposure of HaCaT keratinocytes to three different concentrations (5, 50, and 500 μg/mL) of Rhododendron leaf extracts. Single channel fluorescence, phase contrast and merged micrographs taken with a confocal laser scanning microscope. Violet signals in merged pictures are due to overlapping red, PI-derived signals with blue Draq5™ staining of the nuclei. Cells treated with 0.5 % DMSO served as controls, A) R. hippophaeoides var. hippophaeoides, B) R. minus, C) R. rubiginosum, D) R. cinnabarinum, E) R. ferrugineum, F) R. polycladum, G) R. concinnum, H) R. xanthostephanum, I) R. anthopogon ssp. anthopogon, J) R. ambiguum, K) R. hirsutum, and L) R. racemosum. Bar represents 250 μm. (TIFF 6203 kb) [file 12906_2015_860_MOESM2_ESM.tif]

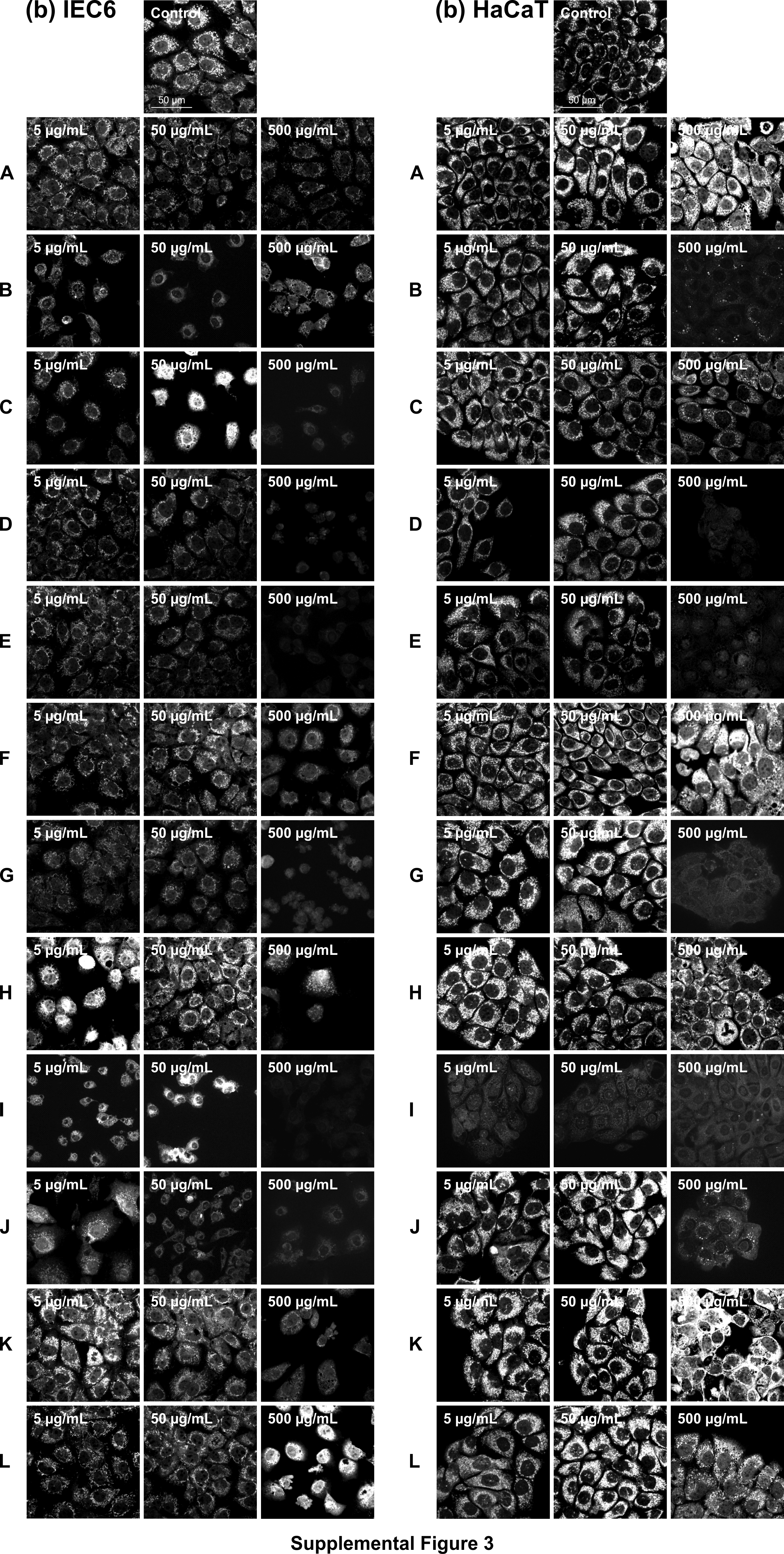

Supplement: Additional file 3: Figure S3. — Overview of mitochondrial morphology in IEC6 and HaCaT cells after a 24h-exposure to three different concentrations (5, 50 and 500 μg/mL) of Rhododendron leaf extracts. Confocal fluorescence images of IEC6 (a, left) and HaCaT (b, right) cells labeled with MitoTracker® Red CMXRos. Cells treated with 0.5 % DMSO served as controls, A) R. hippophaeoides var. hippophaeoides, B) R. minus, C) R. rubiginosum, D) R. cinnabarinum, E) R. ferrugineum, F) R. polycladum, G) R. concinnum, H) R. xanthostephanum, I) R. anthopogon ssp. anthopogon, J) R. ambiguum, K) R. hirsutum, and L) R. racemosum. Bars represent 50 μm. (TIFF 11640 kb) [file 12906_2015_860_MOESM3_ESM.tif]

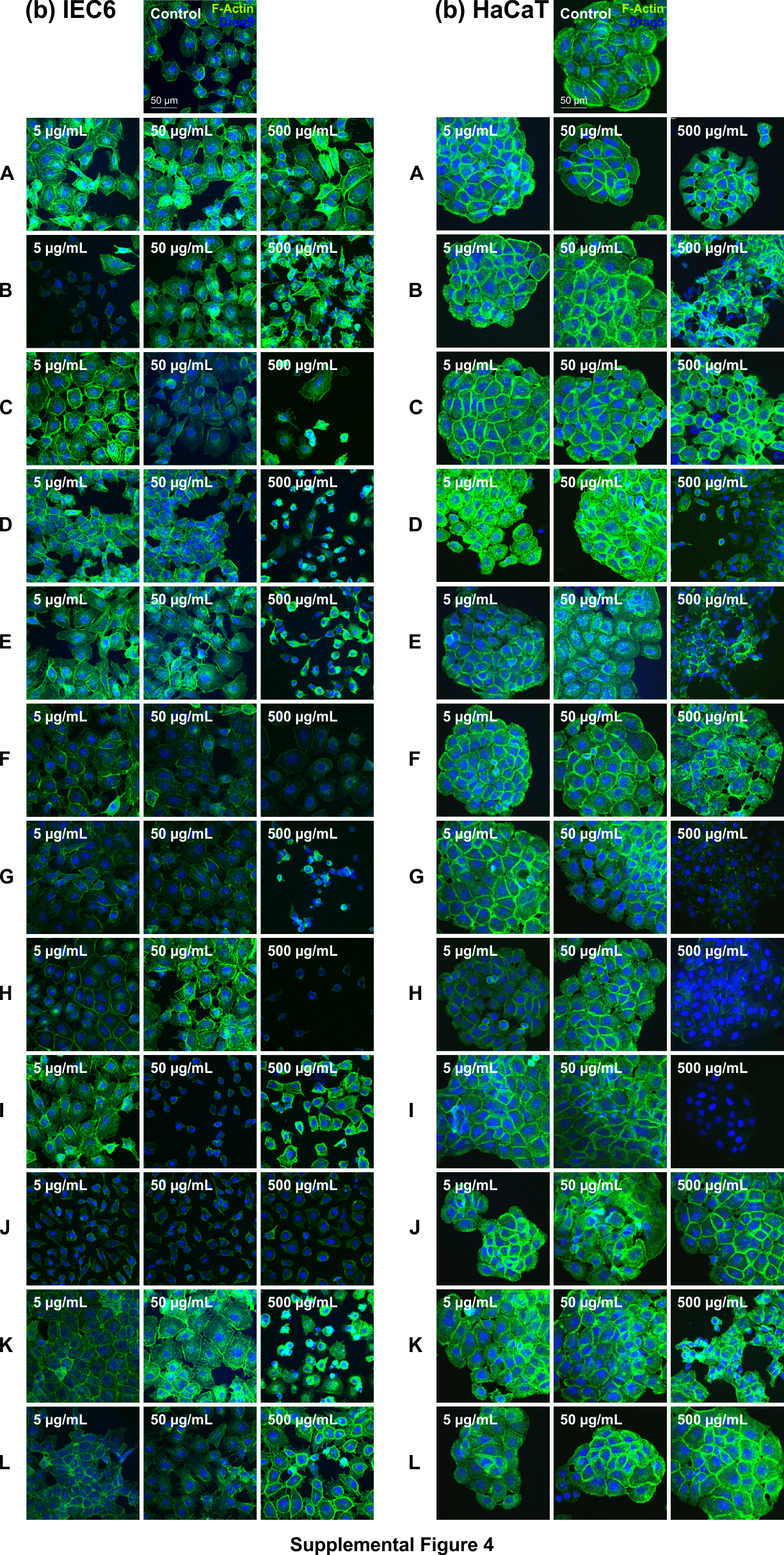

Supplement: Additional file 4: Figure S4. — Overview of the structure of the F-actin system in IEC6 and HaCaT cells after a 24h-exposure to three different concentrations (5, 50 and 500 μg/mL) of Rhododendron leaf extracts. Confocal fluorescence images of IEC6 (a, left) and HaCaT (b, right) labeled with phalloidin (green) and Draq5™ (blue). Cells treated with 0.5 % DMSO served as controls, A) R. hippophaeoides var. hippophaeoides, B) R. minus, C) R. rubiginosum, D) R. cinnabarinum, E) R. ferrugineum, F) R. polycladum, G) R. concinnum, H) R. xanthostephanum, I) R. anthopogon ssp. anthopogon, J) R. ambiguum, K) R. hirsutum, and L) R. racemosum. Bars represent 50 μm. (TIFF 8731 kb) [file 12906_2015_860_MOESM4_ESM.tif]
